# Heterogeneity of Rift Valley fever virus transmission potential across livestock hosts, quantified through a model-based analysis of host viral load and vector infection

**DOI:** 10.1371/journal.pcbi.1010314

**Published:** 2022-07-22

**Authors:** Hélène Cecilia, Roosmarie Vriens, Paul J. Wichgers Schreur, Mariken M. de Wit, Raphaëlle Métras, Pauline Ezanno, Quirine A. ten Bosch

**Affiliations:** 1 INRAE, Oniris, BIOEPAR, Nantes, France; 2 Quantitative Veterinary Epidemiology, Wageningen University and Research, Wageningen, The Netherlands; 3 Wageningen Bioveterinary Research, Lelystad, The Netherlands; 4 Sorbonne Université, INSERM, Institut Pierre Louis d’Epidémiologie et de Santé Publique (IPLESP), Paris, France; University of Warwick, UNITED KINGDOM

## Abstract

Quantifying the variation of pathogens’ life history traits in multiple host systems is crucial to understand their transmission dynamics. It is particularly important for arthropod-borne viruses (arboviruses), which are prone to infecting several species of vertebrate hosts. Here, we focus on how host-pathogen interactions determine the ability of host species to transmit a virus to susceptible vectors upon a potentially infectious contact. Rift Valley fever (RVF) is a viral, vector-borne, zoonotic disease, chosen as a case study. The relative contributions of livestock species to RVFV transmission has not been previously quantified. To estimate their potential to transmit the virus over the course of their infection, we 1) fitted a within-host model to viral RNA and infectious virus measures, obtained daily from infected lambs, calves, and young goats, 2) estimated the relationship between vertebrate host infectious titers and probability to infect mosquitoes, and 3) estimated the net infectiousness of each host species over the duration of their infectious periods, taking into account different survival outcomes for lambs. Our results indicate that the efficiency of viral replication, along with the lifespan of infectious particles, could be sources of heterogeneity between hosts. Given available data on RVFV competent vectors, we found that, for similar infectious titers, infection rates in the *Aedes* genus were on average higher than in the *Culex* genus. Consequently, for *Aedes*-mediated infections, we estimated the net infectiousness of lambs to be 2.93 (median) and 3.65 times higher than that of calves and goats, respectively. In lambs, we estimated the overall infectiousness to be 1.93 times higher in individuals which eventually died from the infection than in those recovering. Beyond infectiousness, the relative contributions of host species to transmission depend on local ecological factors, including relative abundances and vector host-feeding preferences. Quantifying these contributions will ultimately help design efficient, targeted, surveillance and vaccination strategies.

## Author summary

Viruses spread by mosquitoes present a major threat to animal and public health worldwide. When these pathogenic viruses can infect multiple species, controlling their spread becomes difficult. Rift Valley fever virus (RVFV) is such a virus. It spreads predominantly among ruminant livestock but can also spill over and cause severe disease in humans. Understanding which of these ruminant species are most important for the transmission of RVFV can help for effective control. One piece of this puzzle is to assess how effective infected animals are at transmitting RVFV to mosquitoes. To answer this question, we combine mathematical models with observations from experimental infections in cattle, sheep, and goats, and model changes in viremia over time within individuals. We then quantify the relationship between hosts’ viremia and the probability to infect mosquitoes. In combining these two analyses, we estimate the overall transmission potential of sheep, when in contact with mosquitoes, to be 3 to 5 times higher than that of goats and cattle. Further, sheep that experience a lethal infection have an even larger overall transmission potential. Once applied at the level of populations, with setting-specific herd composition and exposure to mosquitoes, these results will help unravel species’ role in RVF outbreaks.

## Introduction

At the beginning of this century, 75% of emerging pathogens in humans were estimated to be zoonotic [1] and 77% of livestock pathogens could be transmitted between different host species [2]. Estimating the relative role different species play in sustaining or amplifying pathogen spread is fundamental for designing control strategies [3–6], yet is hampered by an incomplete understanding of the host(-vector)-pathogen interactions that underlie the spread of these pathogens [7–10].

The potential of a host to contribute to virus transmission is determined by the complex interplay of different factors. For viruses transmitted by arthropod vectors (i.e., arboviruses) these epidemiological interactions are driven both by ecological, population-level factors (i.e., the presence of specific host and vector species and their respective interactions) and the individual-level interactions of the virus with its hosts and vectors. The ability of a host species to infect a susceptible vector upon a potentially infectious contact is determined by the latter. Namely, it derives from i) the viral replication in the host and ii) the ability of a vector to pick up the virus upon blood feeding and subsequently become infected and infectious. While these processes can and have been studied in experimental settings, combining these findings into epidemiologically meaningful parameters is challenging [11–13].

Within-host mathematical models and accompanying inference frameworks have been developed to aid the analysis and interpretation of viral load patterns obtained in controlled infection experiments. Such models provide insights into the biological mechanisms underlying observed patterns [14–18] and how those patterns relate to the clinical expression of the disease [12]. The majority of these modeling efforts are based on viral RNA (or DNA) data, which are indirect measures of infectious virus. Efforts to combine these with infectious virus data (e.g., median tissue culture infectious dose, TCID_50_ or plaque forming units, PFU) have recently emerged for influenza viruses and provide better mechanistic insights into the proportion of particles that are infectious and could contribute to onward transmission [19–24].

Rift Valley fever virus (RVFV) exemplifies the challenges inherent to battling multi-host arboviruses. It was first identified in Kenya, in 1930, after description of an enzootic hepatitis in sheep [25]. The virus has since caused outbreaks throughout the African continent as well as in the Southwest Indian ocean islands (Comoros archipelago, Madagascar) and the Arabian Peninsula [26]. RVFV mainly affects sheep, goats, and cattle, in which it causes abortion storms and sudden death of newborns [27, 28]. Spillover to humans happens through the handling of infectious animal tissue or by vectorial transmission. While most human infections remain asymptomatic or manifest as a mild illness, symptoms can range from flu-like to hepatitis, encephalitis, retinitis and in the most severe cases, haemorrhagic disease [29]. RVFV vector-borne transmission is mainly mediated by *Aedes* and *Culex* spp. mosquitoes, making its establishment possible in a wide range of ecosystems [30]. While sheep are generally believed to be the most important host species [31–33], efforts fall short of quantifying livestock hosts’ relative contribution to RVFV transmission.

Here, we aim to gain more insight into the relative importance of livestock species in RVFV transmission. Using experimental data and mathematical modeling, we derive estimates of hosts’ individual potential to transmit RVFV to vectors during their infectious period.

## Results

### Overall approach

**Fig 1:**
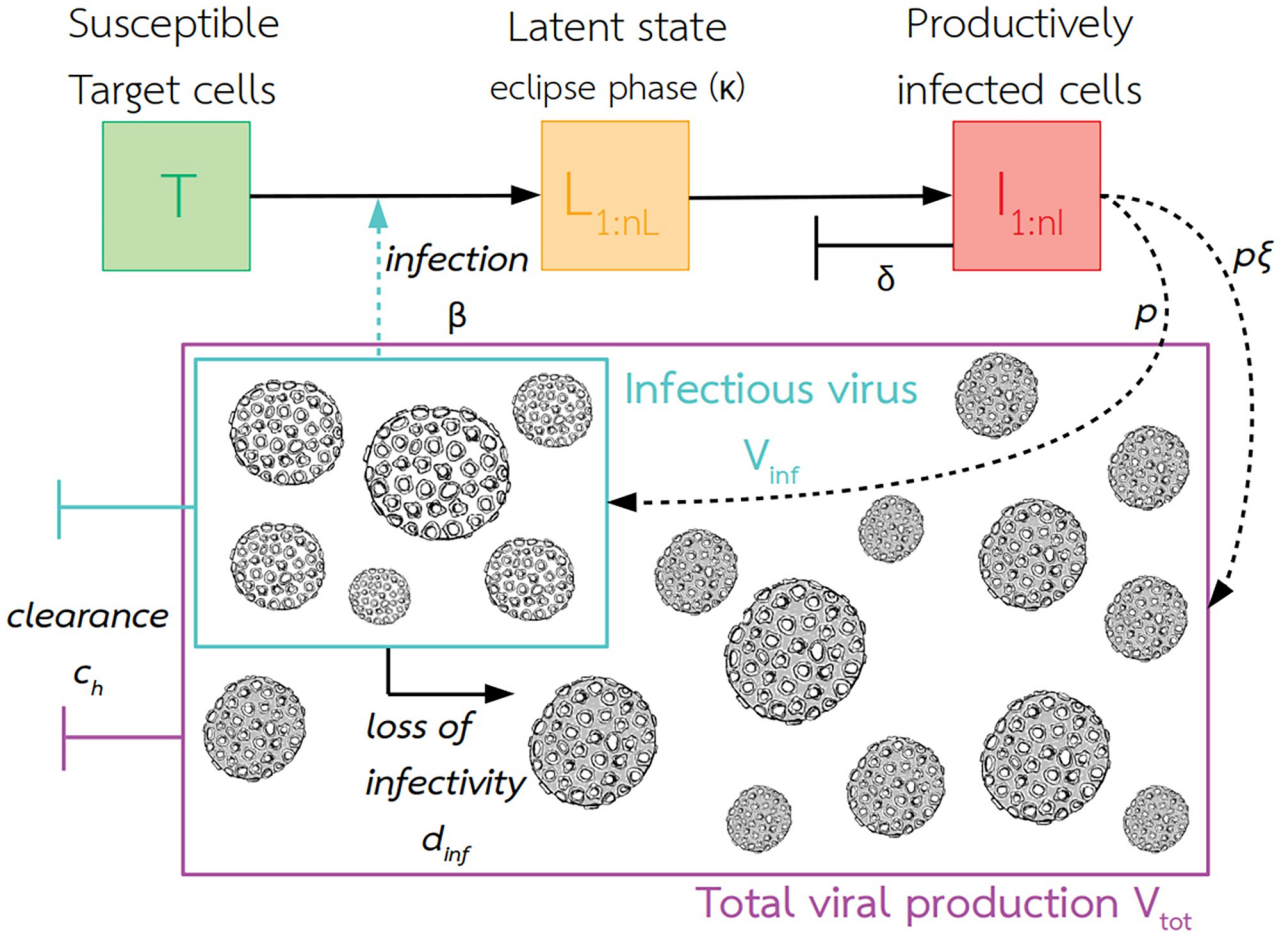
Graphical representation of the within-host model. Infectious viruses *V*_*inf*_ were fitted to TCID_50_ measures, and total viral production *V*_*tot*_ to RT-qPCR measures. The eclipse phase (state *L*) is the period between the infection of a cell by a virus and the presence of mature viruses within the cell. Productively infected cells *I* are the only ones producing progeny virions. Subscripts in *L* and *I* indicate the use of Erlang distributions for the time spent in those states. Target cells are not replenished and only productively infected cells die. Model assumptions, equations and parameter definitions can be found in Materials and methods, Eq (1), and Table 1.

We developed a mechanistic compartmental within-host model, representing the infection of target cells and the subsequent production of viral particles, not all of which are infectious (Fig 1). We distinguished the total amount of viral particles produced by infected cells, *V*_*tot*_, and the subpart capable of infecting new cells, *V*_*inf*_. We fitted this model to time-series of viral RNA (RT-qPCR) and infectious virus (TCID_50_), measured daily in calves (n = 8), lambs (n = 16), and young goats (n = 8) intravenously inoculated with a virulent RVFV strain ([34], Materials and methods). We compared the cell-level basic reproduction number *R*_0_ and mean generation time *T*_*g*_, between groups. We quantified the relationship between vertebrate hosts’ infectious titers and transmission to mosquitoes using data we extracted through a systematic literature review. Finally, we estimated the net infectiousness of livestock species, a metric proportional to the number of mosquitoes a host would infect over the entire course of its infection.

### Data description

All animals became viremic. In total, 10/16 lambs succumbed to the infection or were euthanized, 3 to 7 days after RVFV inoculation, while others survived until the end of the experiment (2 weeks). All calves and young goats survived until the end of the experiment. Animals reached their maximum RNA levels (average 8.79 log_10_ copies/ml, standard deviation 0.81 log_10_ copies/ml) and infectious titers (average 5.16 log_10_TCID_50_/ml, standard deviation 1.16 log_10_TCID_50_/ml) on day 2 or 3 post-infection.

### Within-host model of RVFV infection

**Fig 2:**
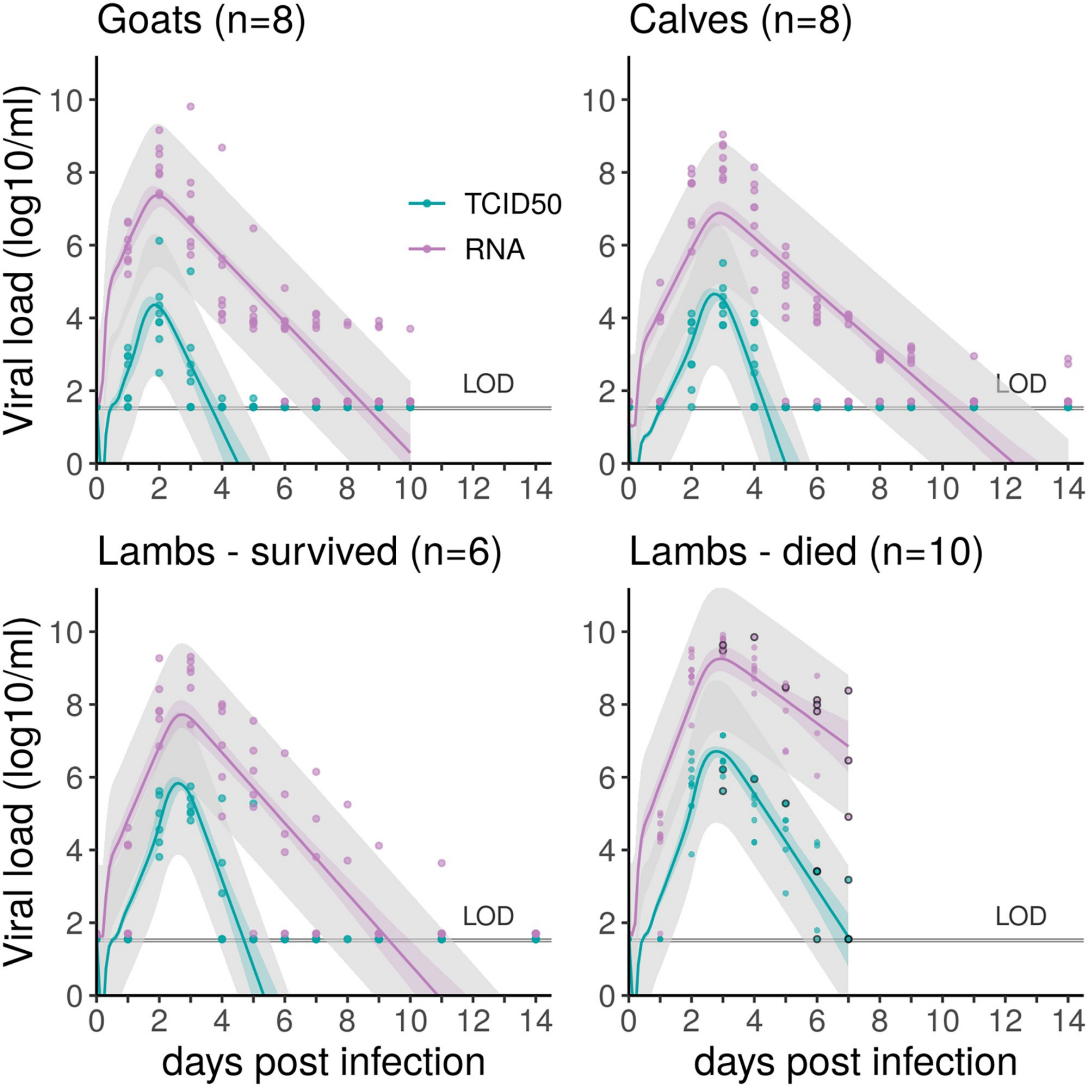
Data on viral RNA (RT-qPCR) and infectious virus (TCID_50_), in log_10_/ml of plasma, and model fits, for host groups showing significantly different viral dynamics. Circles are data points. Solid colored lines show the median fit, obtained from 1000 posterior draws. Inner envelopes using the same colors shows the uncertainty from the parameter estimation process (quantiles [2.5–97.5]% of these posterior draws). Outer grey envelopes show the 95% uncertainty bounds associated with the observation process. We assumed this to be normally distributed with a standard deviation of 1 (log10 scale), in line with the sampling error. Purple is for viral RNA and blue for infectious viruses. For lambs which died from RVF, circled points represent individuals’ time of death. LOD = limit of detection, 1.55 for TCID_50_, 1.7 for viral RNA (log_10_).

We fitted a within-host model to four datasets, measuring viral RNA and infectious virus in RVFV-infected lambs (surviving; dying), calves, and young goats, using a Bayesian framework (Materials and methods). The model consisted of 10 parameters, 5 of which were held constant (Table 1). We estimated the death rate *δ* of infected cells, their total daily production of viral particles *ξp*, among which *p* are infectious, the degradation rate *d*_*inf*_ of infectious viruses into non-infectious viruses, and the clearance rate *c*_*h*_ of viral particles. Parameter values were then used to calculate the cell-level basic reproduction number *R*_0_ and mean generation time *T*_*g*_. Initial conditions were set using elements of the experimental protocol along with a sensitivity analysis (Materials and methods, Table 1). Outputs from the Markov Chain Monte Carlo (MCMC) procedure can be found in Section S.1.1 in S1 Text. The fits satisfyingly capture the dynamics present in the data (Fig 2).

**Table 1:** Parameters of the within-host model. Values if fixed, prior range (uniform distribution) if estimated.

Name	Meaning	Value/Estimated	Reference/Prior
*T*_0_	initial number of susceptible target cells	Fixed within MCMC, estimated *a priori* through likelihood profiles	see Fig A in S1 Text
*L*_0_	initial number of cells in latent state	0	
*I*_0_	initial number of productively infected cells	0	
*V*_*inf*,0_	initial number of infectious virions	12.5 for calves, 62.5 for goats, 52.6 for lambs (per ml of plasma, total inoculum per animal being 10^5^)	References for plasma:body weight ratios [35–37]
*β*	rate governing infection of target cells by infectious virions	set such as *βT*_0_ = 48 day^-1^	assumed
*n*_*L*_, *n*_*I*_	number of *L* and *I* states for the Erlang distributions	20	[38, 39]
*κ* ^−1^	eclipse phase duration	1/3 day (8 hours)	P. Wichgers-Schreur personal communication, observed *in vitro*
*δ*	death rate of productively infected cells	Estimated	[0.1; 10]° day^-1^
*p*	rate of production of infectious virions	Estimated	[0.2; 3.10^4^]^†^ day^-1^
*d*_*inf*_	rate of degradation of infectious virions into non-infectious viral particles	Estimated	[0.1; 10] day^-1^
*c*_*h*_	host-driven clearance rate	Estimated	[0.1; 10] day^-1^
*σ*	correction factor to convert from infectious virions (plaque forming units) to TCID_50_	0.69	[40]
*ξ*	ratio of total viral particles to infectious virions, as produced by infected cells	Estimated	[1; 1000]^†^ day^-1^

^†^: these values were applied for each *L* (respectively *I*) states, so a daily rate per *L* (*I*) cell (not state) can be obtained by multiplying by *n*_*L*_ (*n*_*I*_)

°: *δ* was constrained to be inferior to *κ* and (*c*_*h*_ + *d*_*inf*_), as advised by [41].

**Fig 3:**
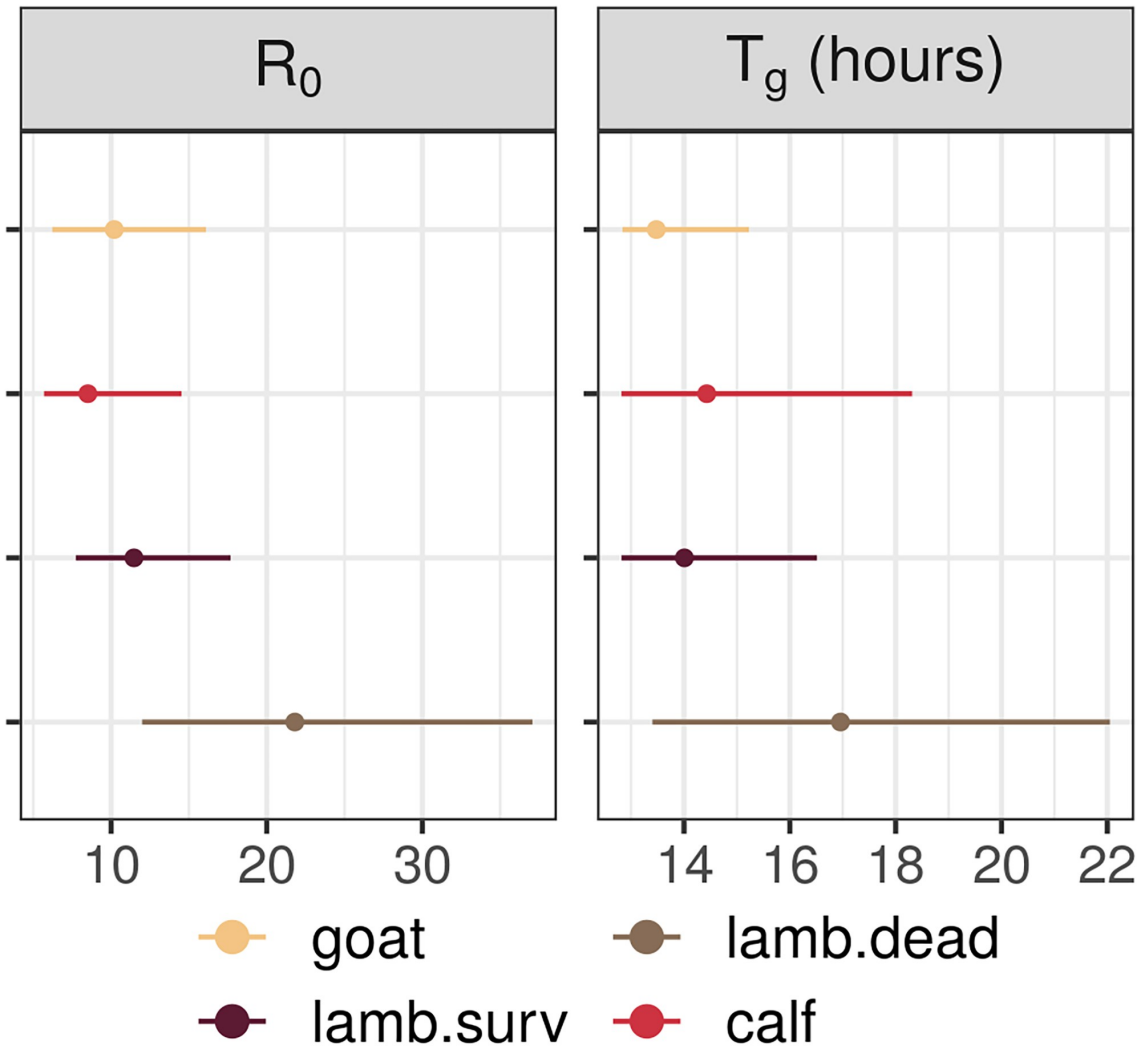
Outcome measures per host group. Points are median estimates, lines show highest density intervals, computed from joint posterior distributions (3 chains). Basic reproduction number *R*_0_ is computed with Eq (3) and generation time *T*_*g*_ with Eq (4). Note that generation times are constrained in their lower values due to the eclipse phase duration (*κ*^−1^) and rate of virus entry into cells (*β*) being fixed (Table 1).

The model selection performed highlights different viral load dynamics between livestock species (Deviance Information Criterion (DIC) 1307 *vs* 1186, comparison based on surviving animals as calves and young goats all survived, Fig 2). In particular, the ratio of daily viral RNA over infectious viruses produced (*ξ*) is the highest in the goat group, meaning that the replication process might be less efficient in this species (Table 2). The highest density intervals (HDIs) for this parameter are wide (Table 2), but the posterior distributions remain informative, as knowledge was gained compared to uniform prior distributions (Fig D in S1 Text). In addition, among surviving hosts, the lifespan of infectious particles (*d*_*inf*_ + *c*_*h*_)^−1^ is estimated to be the longest in goats (Table 2). The resulting dynamics show viremia in goats peaks sooner than in calves and in lambs, but with a lower peak value for infectious viruses (Fig 2). Lambs have on average the most infectious viral particles. Model results indicate this could be a result of a slightly higher daily production rate *p* (Table 2), as well as their initial susceptible cell population, which we estimated to be higher than in other species (Fig A in S1 Text). Characterizing the infectious replication process through the basic reproduction number *R*_0_ and generation time *T*_*g*_ (Materials and methods, Eqs (3) and (4)) shows no strong differences between species when comparing surviving individuals (Fig 3). *R*_0_ ranges from 8.51 (median; 95% HDI 5.69—14.53) for calves, to 11.47 (median; 95% HDI 7.73—17.68) for lambs. *T*_*g*_ (i.e., the time between infection of a cell and infection of a secondary cell) ranges from 13.48h (median; 95% HDI 12.84h—15.23h) in goats to 14.43h (median; 95% HDI 12.82h—18.31h) in calves.

**Table 2:** Parameter estimates per host group. Median of joint posterior distributions (3 chains) and HDI = highest density interval (95%). All parameters are in unit day^-1^, see Materials and methods and Table 1 for detailed definitions. The HDI is built such as every point inside the interval has higher credibility than any point outside the interval [42].

		Estimate: median [HDI]			
	Parameter	Goat	Calf	Lamb surv.	Lamb dead
*δ*	*I* death rate	2.61 [1.91; 3.0]	2.17 [1.30; 3.0]	2.34 [1.60; 3.0]	1.52 [0.85; 2.44]
*p*	production of *V*_*inf*_	20.14 [13.33; 29.40]	14.98 [12.31; 17.89]	21.53 [17.09; 26.50]	25.27 [19.72; 31.00]
*ξ*	ratio $\frac{V_{tot}}{V_{inf}}$ produced	672.76 [333.96; 999.56]	75.16 [20.60; 161.05]	44.33 [9.66; 104.48]	221.15 [50.57; 510.97]
*d*_*inf*_	degradation *V*_*inf*_ → *V*_*tot*_	2.10 [1.36; 2.92]	3.77 [2.17; 6.63]	3.26 [2.19; 4.48]	1.60 [0.90; 2.31]
*c*_*h*_	clearance of *V*_*inf*_ and *V*_*tot*_	2.06 [1.88; 2.24]	1.72 [1.53; 1.92]	2.24 [1.94; 2.53]	1.43 [0.87; 2.01]

Among lambs, individuals succumbing to RVF are characterized by higher viral loads, both total and infectious, and a slower decay after the peak is reached (Fig 2). The best model fit is achieved when allowing parameters to vary depending on the survival of the individuals (Fig 2, DIC 928 vs 745), indicating significantly different within-host dynamics depending on clinical outcome. In particular, we estimated that both infected cells and infectious viral particles have prolonged lifespans in dying lambs (*δ*^−1^ and (*d*_*inf*_ + *c*_*h*_)^−1^ respectively, Table 2). This impacts *R*_0_ which is 1.88 times higher (median ratio; 95% HDI 0.84; 3.51) in dying individuals than surviving ones, and *T*_*g*_, which is 1.19 times longer (median ratio; 95% HDI 0.91; 1.65) in dying individuals than surviving ones. Besides, the ratio of daily viral RNA over infectious viruses produced (*ξ*), which does not influence *R*_0_, is higher in dying lambs than surviving ones (Table 2).

### Dose-response relationship in RVFV mosquito vectors

Through a systematic review, we identified 9 papers from which data could be extracted to estimate the relationship between vertebrate host infectious titers and associated infection rates in vectors (Materials and methods, Section S.2.1 in S1 Text). Selected experiments were performed with hamster hosts, *Aedes* or *Culex* spp. vectors, using RVFV strain ZH501.

**Fig 4:**
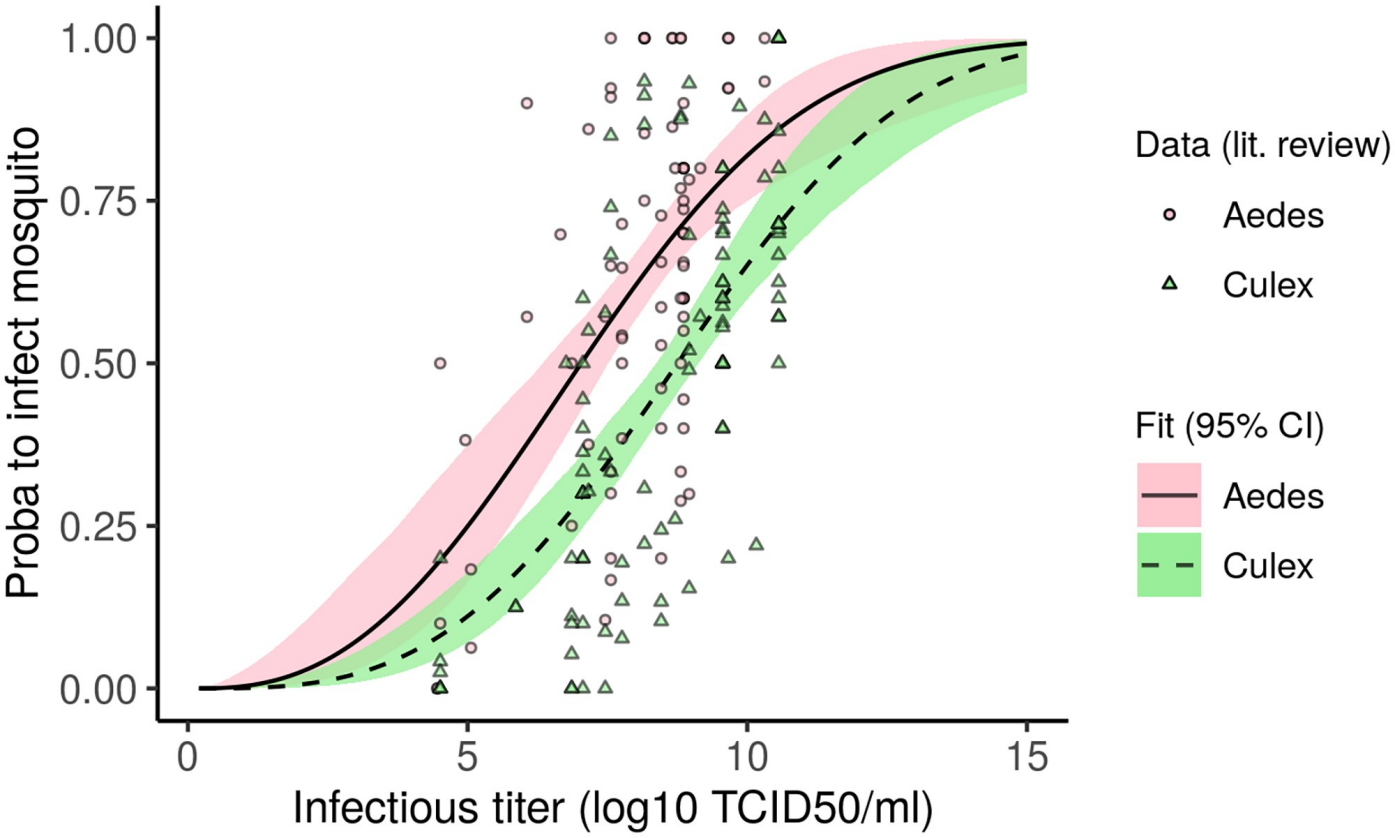
Dose-response relationships linking host infectious titers to the probability to infect mosquito vectors. Data retrieved from a systematic review (Materials and methods, Section S.2.1 in S1 Text). Points and triangles show infection rates (presence of RVFV in mosquito bodies, legs excluded) from experiments performed with hamsters with RVFV strain ZH501. Fits were obtained with Eq (S.6) in S1 Text using a betabinomial likelihood to account for overdispersal in the data. Confidence intervals result from 1000 replicate trajectories. Note that infectious titers >10 log_10_ TCID_50_/ml are not to be expected in hosts, but were included to show the full curve.

Dose-response curves differ significantly between *Aedes* and *Culex* spp. (Fig 4, Section S.2 in S1 Text). At 5 log_10_ TCID_50_/ml for instance, which most animals could reach or exceed (Fig 2), there is 25% [17; 37] probability to infect an *Aedes* spp. vector and 11% [7; 18] probability to infect a *Culex* spp. vector (point estimate and 95% confidence interval, Fig 4). We did not find a significant effect of temperature and number of days post-exposure on infection rates (Sections S.2.2, S.2.3 in S1 Text). The effect of dose is best captured by Eq (S.6) in S1 Text, used by [43], fitted with a betabinomial likelihood accounting for overdispersal in the data (Section S.2.3 in S1 Text). Species-specific curves were estimated for *Aedes vexans*, *Aedes japonicus*, *Culex nigripalpus*, and *Culex tarsalis* (Section S.2.3 and Fig G in S1 Text). While there is intra-genus variability, infection rates in *Aedes vexans* and *Aedes japonicus* are on average higher than in *Culex nigripalpus*, and *Culex tarsalis* at similar host infectious titers (Fig G in S1 Text).

### Net infectiousness of RVFV livestock hosts

**Fig 5:**
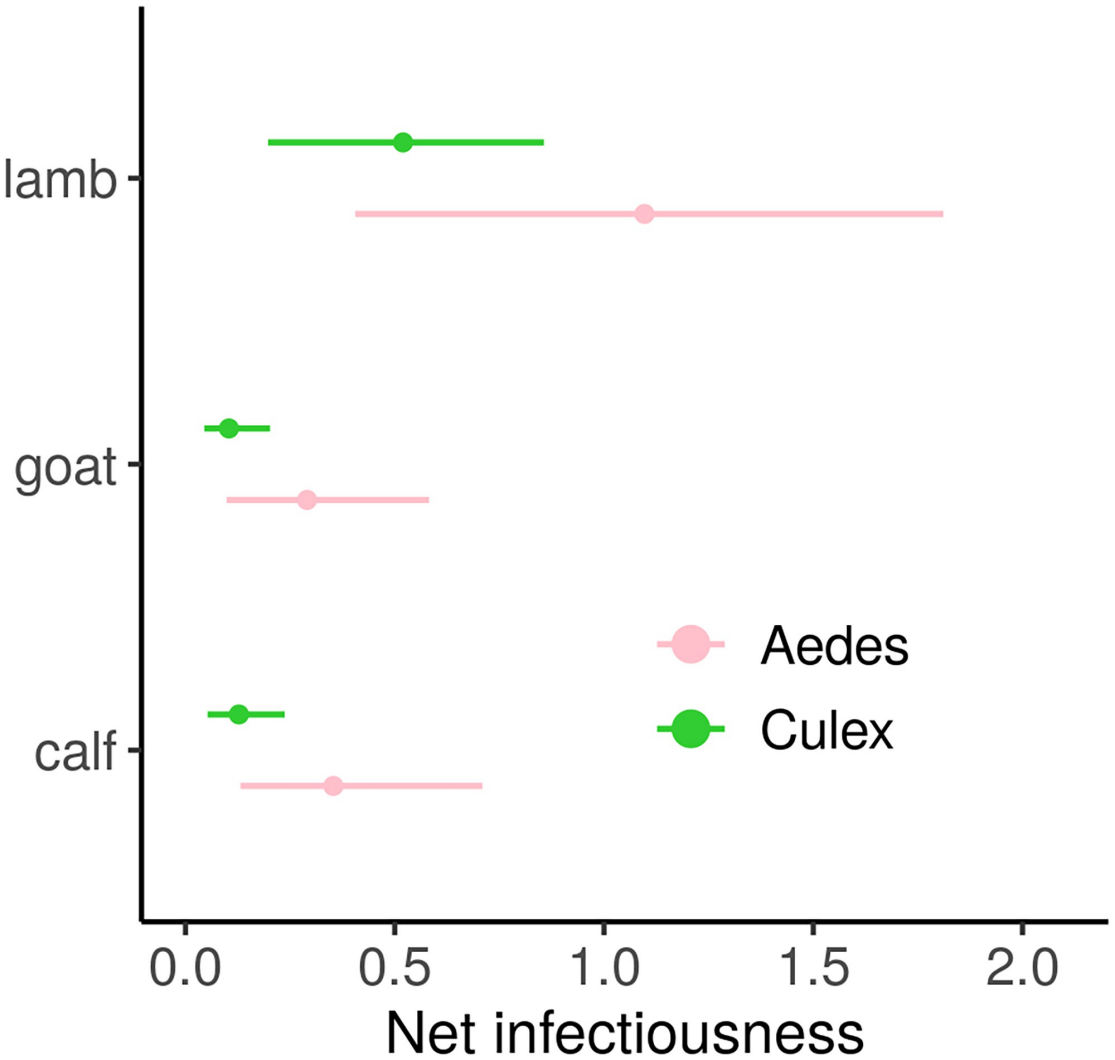
Net infectiousness of RVFV livestock host species, function of the mosquito genus involved in transmission. Points are median estimates, lines show highest density intervals, computed using 1000 parameter sets from the within-host model and dose-response curve respective fitting procedures. For lambs, parameters were sampled in the posteriors of both surviving and dying groups, according to the survival rate observed in the original dataset (6/16, Fig H in S1 Text). Time of death also varied according to a Weibull survival model (Materials and methods).

Net infectiousness (NI, Eq (5)) varies with both host species and mosquito genus involved (Fig 5). NI is lowest for goats and highest for lambs. The relative differences in NI between host species is stronger when comparing transmission to *Culex* (median ratio lamb:goat 4.79; median ratio lamb:calf 3.75) than to *Aedes* mosquitoes (median ratio lamb:goat 3.65; median ratio lamb:calf 2.93). Every host type studied has the highest NI when bitten by an *Aedes* spp. vector, but the uncertainty around NI estimates decreases when considering *Culex* bites (Fig 5).

Lambs’ NI varies with the expected death rate among lambs (Materials and methods, Fig H in S1 Text). Lambs dying from RVF have a higher NI than lambs surviving (Fig H in S1 Text). Indeed, dying lambs are more infectious than their surviving counterparts during their whole viremic period, which in 60% of cases can last longer (day 7) than the infectious period of surviving individuals (probability < 1% to infect an *Aedes* or a *Culex* past day 5 post-inoculation). When bitten by an *Aedes* spp. vector, lambs NI ranges from 0.24 to 2.54, increasing by a factor 1.93 (median ratio) from surviving to succumbing individuals. When bitten by a *Culex* spp. vector, lambs NI ranges from 0.12 to 1.36, increasing by a factor 2.22 (median ratio) from surviving to succumbing individuals.

## Discussion

We have presented the results of a data-driven estimation of livestock hosts RVFV transmission potential, providing mechanistic insights into potential sources of heterogeneity between species. Our results demonstrate that sheep are the most infectious livestock hosts, and that virulent infection leading to death reinforces the infectiousness of this species. We also showed that in the current literature, lower infectious doses are needed on average to infect *Aedes* spp. vectors than *Culex* spp.. The framework presented here can be applied to other multi-host arboviruses to estimate transmission potential, a key component of hosts contribution to transmission at large scale.

The suite of experimental data used in our study incorporated the major elements needed for an epidemiologically relevant estimation of hosts’ transmission potential. We included both viral RNA and infectious viruses, measured *in vivo*, in natural RVFV hosts. Similar existing models used data coming either from *in vitro* experiments [19, 20, 23, 24, 44], or from model hosts, such as ferrets for influenza [21, 22]. The breeds infected in our dataset, which are dominant breeds from Europe, make our estimates directly relevant for scenarios of RVFV emergence on this continent [45]. A comparison with African breeds is required to know if the relative differences in infectiousness are maintained. Heterogeneity among RVFV strains should also be studied [46]. Performing infection through mosquito bites rather than intravenous injection would ensure a natural course of infection, although the protocol presently used was shown to yield similar viral load dynamics as mosquito-mediated infection [47]. This would further allow for the exploration of the impact of heterogeneity of exposure (i.e., number of infectious bites or infectious titers in vector saliva) on infectiousness. Quantifying more precisely the effect of aging on animals’ viral dynamics and pathogenesis is needed to complete our results [48]. Finally, measuring human viral loads, as early as possible post-infection, will be key to complete our understanding of hosts’ contribution to RVFV transmission.

Our within-host model is the second developed for RVFV [49], but the first to mechanistically represent the process of viral production from host cells. This enabled an identification of processes driving differences between groups and an increased understanding of the cell-level viral replication process. First, we estimated a less efficient replication in goats, further advocating for the use of infectious virus measures in order not to overestimate transmission potential [50]. Besides, we estimated the lifetime of infectious viral particles and infected cells to be longer in dying lambs than their surviving counterparts, which calls for an exploration of corresponding (immune) mechanisms in future experiments. The uncertainty around parameter estimates remains important, and summarizing parameter estimates into aggregated outcome measures *R*_0_ and *T*_*g*_ put those mechanistic differences into perspective. Indeed, once correlations between parameters are taken into account, the replication process is most different between severe and moderate infection within sheep and less so between host species. The model could be refined by incorporating an explicit immune response [51, 52] or taking into account the genomic composition of viral particles [53, 54], but the quantity of information needed (number of timesteps and replicates, inclusion of data on immune responses) could hamper this costly data collection. Alternatively, routinely collected data such as body temperature could constitute an interesting lead to explore time-varying parameters, as a proxy of the immune response. Dedicated modeling would first be needed to determine i) the form of the relationship between temperature and the immune response, most likely with cytokines [55, 56], and ii) which model parameters would be impacted by such an immune response.

By gathering relevant competence studies into a meta-analysis, we quantified the relationship between infectious titers and mosquito RVFV infections. To our knowledge, such dose-response relationship had not been quantified for RVFV. This results in a lack of precision in between-host RVFV transmission models which usually assume constant infectiousness of hosts over their infectious period. Quantifying how the probability to infect a vector increases with dose will also affect the stochasticity of transmission in small populations (be it emergence or extinction). Dose-response curves have been important for the study of other arboviruses, e.g., for exploring the role of asymptomatic dengue infections [12] or the epidemic potential of *Aedes albopictus* for Zika virus [57]. One important originality of our work was to highlight a higher susceptibility of *Aedes* spp. vectors to RVFV infection compared to *Culex* spp. vectors, at similar infectious titers. Further studies are needed to confirm whether this higher probability of infection is also accompanied by a higher probability of the mosquito becoming infectious itself. This would require the detection of infectious particles in mosquitoes’ saliva, which was only performed in 23 out of 185 data points in the present systematic review.

A lot remains unknown about the bottlenecks of arboviruses propagation in mosquitoes [58]. It can depend on species within each genus [59, 60] or even mosquito provenance (field *vs* laboratory-reared, [61–63]), in part because of the role of temperature [64]. Further experiments are needed to know whether a given infectious titer sampled during the increasing or the decreasing phase of viral dynamics would yield the same probability to infect vectors. This comes down to defining what makes a viral particle infectious to host cells *vs* vector cells, and might relate to the efficiency of genome packaging by those cells [54]. Mechanistic modeling will help grasp the complexity of involved processes.

Our study provided key estimates of RVFV livestock hosts’ transmission potential. It quantified for the first time the prominent role of sheep, which are 3 to 4 times more infectious than cattle and goats, due to more infectious viruses and a longer infectious period. In addition, fatal infection in sheep does not diminish transmission potential but could rather increase it, based on time of deaths observed in our dataset. This entails that most vulnerable populations, in addition to suffering more deaths, will likely experience larger outbreaks.

Understanding the relationship between infectiousness and pathogen load represents a key challenge to connect modeling scales [65]. We have importantly contributed to deciphering this relationship for Rift Valley fever virus. Combining these results with ecological factors such as vector presence, population dynamics, and trophic preference, as well as human factors, which define the presence of livestock hosts and their mobility, will increase our understanding of RVFV transmission dynamics at large scale. These interacting scales might yield unexpected patterns and reshape the way we design surveillance and control strategies for multi-host arboviruses in general.

## Materials and methods

### Ethics statement

The animal experiment was conducted in accordance with European regulations (EU directive 2010/63/EU) and the Dutch Law on Animal Experiments (Wod, ID number BWBR0003081). Permissions were granted by the Dutch Central Authority for Scientific Procedures on Animals (Permit Number:AVD4010020185564). All experimental protocols were approved by the Animal Ethics Committees of Wageningen Research.

### Experimental design

Data on viral RNA and infectious viruses were obtained from a published study on a candidate RVFV vaccine [34]. Mock vaccinated animals (8 lambs, 8 calves, 8 young goats) were inoculated intravenously with 5 log_10_ TCID_50_ of strain rRVFV 35/74. Plasma was sampled daily for 10 days in goats, daily for 9 days then every two days until day 14 in calves and lambs. Animals’ age was 2–3 weeks for calves, 8–10 weeks for lambs and goats. The average body weight of animals, used further to calibrate the inoculum per ml of plasma, was 45 kg for lambs, 30 kg for goats, and 80 kg for calves. Animals were purchased from conventional Dutch farms, and the breed was Texel cross for sheep, Saanen for goats, and Holstein-Friesian for cattle [34]. An additional dataset obtained from 8 lambs, following the same protocol, was added.

Viral RNA was isolated with the NucliSENS easyMAG system according the manufacturer’s instructions (bioMerieux, France) from 0.5 ml of plasma. Briefly, 5 *μ*l RNA was used in a RVFV RT-qPCR using the LightCycler one-tube RNA Amplification Kit HybProbe (Roche, Almere, The Netherlands) in combination with a LightCycler 480 real-time PCR system (Roche) and the RVS forward primers (AAAGGAACAATGGACTCTGGTCA), the RVAs (CACTTCTTACTACCATGTCCTCCAAT) reverse primer and a FAM-labelled probe RVP (AAAGCTTTGATATCTCTCAGTGCCCCAA). Virus isolation was performed on RT-qPCR positive samples with a threshold above 10^5^ RNA copies/ml as this has been previously shown to be a cut-off point below which no live virus can be isolated. For the virus isolations, plasma was used. Briefly, BHK-21 cells were seeded at a density of 20,000 cells/well in 96-well plates. Serial dilutions of samples were incubated with the cells for 1.5h before medium replacement. Cytopathic effect was evaluated after 5–7 days post-infection and tissue culture infective dose 50 (TCID_50_) was calculated using the Spearman-Kärber algorithm.

### Within-host model of RVFV infection

Our mechanistic model (Fig 1) is formulated as a set of ordinary differential equations, and is similar to existing within-host models developed for influenza [21, 24]:

$$\begin{array}{c} \begin{array}{ccc} \frac{dT}{dt} & =-\beta TV_{inf} & \\ \frac{dL_{1}}{dt} & =\beta TV_{inf}-n_{L}\kappa L_{1} & \\ \frac{dL_{i}}{dt} & =n_{L}\kappa \left(L_{i-1}-L_{i}\right), & i=2,...,n_{L}\\ \frac{dI_{1}}{dt} & =n_{L}\kappa L_{n_{L}}-n_{I}\delta I_{1} & \\ \frac{dI_{j}}{dt} & =n_{I}\delta \left(I_{j-1}-I_{j}\right), & j=2,...,n_{I}\\ \frac{dV_{inf}}{dt} & =p\sum_{j=1}^{n_{I}}I_{j}-d_{inf}V_{inf}-c_{h}V_{inf}-\sigma \beta TV_{inf} & \\ \frac{dV_{tot}}{dt} & =\xi p\sum_{j=1}^{n_{I}}I_{j}-c_{h}V_{tot} \end{array} \end{array}$$ (1)

In this model, infectious viruses *V*_*inf*_ infect susceptible target cells *T* at rate *β*. Infected cells first go through a latent state, *L* (eclipse phase). Then, they become productively infected cells, *I*. These cells produce viral particles *V*_*tot*_ at rate *ξp*, not all of which are infectious (*V*_*inf*_ produced at rate *p*). Infectious viruses degrade into non-infectious viruses at rate *d*_*inf*_, which does not impact total viral production *V*_*tot*_. A similar host clearance rate *c*_*h*_ is applied to both non-infectious and infectious particles.

To achieve realistic distributions of time spent in *L* and *I* states, we used Erlang distributions. This means that infected cells go through *n*_*L*_ latent stages and *n*_*I*_ infectious stages, where the time spent in each stage is exponentially distributed. We used *n*_*L*_ = *n*_*I*_ = 20, sufficient for the resulting latent and infectious periods to be almost normally or lognormally distributed [38, 39]. The mean of these Erlang distributions are *κ*^−1^ and *δ*^−1^, and their variance $\frac{1}{n_{L}\kappa^{2}}$ and $\frac{1}{n_{I}\delta^{2}}$.

We used a target-cell limited model, meaning that the depletion of target cells is what triggers the viral load peak and subsequent decline. We did not incorporate an explicit immune response. However, as explained by [66], this type of model can be seen as equivalent to assuming a constant effect of the immune response (IR). This IR can act implicitly by limiting the number of cells susceptible to the infection, removing infected cells or viral particles.

We fitted *V*_*inf*_ to TCID_50_ measures and *V*_*tot*_ to RT-qPCR measures. As TCID_50_ measures the dose needed to induce a cytopathic effect in 50% of the cells, a conversion factor *σ* is needed to express it as a quantity of infectious viruses, usually measured in plaque forming units (PFUs). Here, we set *σ* = 0.69 TCID_50_/ml, consistent with 1 ml virus stock having half the number of (PFUs) as TCID_50_ using Poisson sampling [40].

We used a Metropolis Rosenbluth Monte Carlo Markov Chain (MCMC) algorithm to fit our model, implemented in R, using the *odin* package (https://github.com/mrc-ide/odin) to speed up simulations. In our composite log-likelihood *f* (Eq (2)), we assumed log_10_ viremia measurements had normally-distributed errors. Below, *φ* and *ϕ* are respectively the probability and cumulative density functions of the normal distribution, and *ϵ*^2^ is taken to be 1 [14]. *D* is the measure of either viral RNA or infectious viruses (subscript *i*), and *x* the associated model prediction (either *V*_*tot*_ or *V*_*inf*_). Measures below the limit of detection (LOD) are considered to be at or below LOD [14]. The final log-likelihood of a given model parametrization is the sum across types of measures *i*, timesteps *j*, and individuals *k* of the group under consideration.

$$\begin{array}{c} \begin{array}{cc} f & =\sum_{i,j,k}{\text{log}}_{10}[\varphi {\left({\text{log}}_{10}\;D_{i,j,k}|{\text{log}}_{10}\;x_{i,j,k},\;\epsilon^{2}\right)}^{1-c_{i,j,k}}\;\phi {\left({\text{log}}_{10}\;LOD_{i}|{\text{log}}_{10}\;x_{i,j,k},\;\epsilon^{2}\right)}^{c_{i,j,k}}]\\ c_{i,j,k} & =0\;\text{if}\;D_{i,j,k}>LOD_{i}\text{,}\;\text{else}\;c_{i,j,k}=1\\ & {\text{log}}_{10}\left({\text{LOD}}_{\text{RNA}}\right)=1.7\\ & {\text{log}}_{10}\left({\text{LOD}}_{\text{TCID50}}\right)=1.55 \end{array} \end{array}$$ (2)

The score *f* obtained at each iteration was used by the algorithm to determine if a parameter set should be accepted. At each iteration, parameters were simultaneously sampled using normal distributions centered around their last accepted value, with a standard deviation specific to each parameter. To obtain acceptance rates between 10% and 45% (the optimal acceptance rate being 23.4% as shown by [67]) for each parameter, we used a custom function which determines appropriate standard deviations for their sampling. Fixed and estimated parameters can be found in Table 1, chosen in agreement with identifiability analyses of similar models [66, 68]. Priors represent the probability distribution of possible parameter values, based on prior knowledge. We used uniform distributions, with bounds intended to allow a wide exploration of parameter values while being biologically realistic.

Our fitting procedure worked as follows: for each dataset to fit, we ran small chains (10,000 iterations, 5,000 burn-in period) fixing *T*_0_ at different values spread across [3;6.5] log_10_/ml plasma. The best *T*_0_ value was then assessed through maximum log-likelihood profiles (Fig A in S1 Text) and kept for longer chains. Three long chains were run (100,000 iterations, 20,000 burn-in) for each dataset. The Gelman Rubin diagnostic test was used to assess common convergence of the chains (Fig C in S1 Text). Correlation between estimated parameters was assessed (Fig E in S1 Text).

To determine whether viral load dynamics *V*(*t*) differ between livestock host groups, we ran the inference procedure in two distinct ways: treating these groups as equal (aggregating datasets) or different (fitting done for each dataset separately, Section S.1.1 in S1 Text). The resulting joint posterior distributions were used to compute the Deviance Information Criterion (DIC) of these models and select those with the smallest DIC (Section S.1.1 in S1 Text). We did not attempt to find differences between individuals of a given group.

To characterize the replication process at the beginning of the infection, we computed two outcome measures from the parameters of our model. The basic reproduction number *R*_0_ (Eq (3), [24, 69]) is defined as the average number of new infected cells produced by one infected cell introduced into an entirely susceptible target-cell population. The generation time *T*_*g*_ (Eq (4), [24, 70, 71]) is the average time between the infection of a cell and the infection of a secondary cell, again in an entirely susceptible target cell population. The formula for *T*_*g*_ was adapted to a model using Erlang distributions (for time spent in *L* and *I* states). How it changes compared to *T*_*g*_ computed for models with exponential distributions is explained in Section S.1.2 in S1 Text.

$$\begin{array}{c} R_{0}=\frac{\beta T_{0}p}{\delta (c_{h}+d_{inf}+\sigma \beta T_{0})} \end{array}$$ (3)

$$\begin{array}{c} T_{g}=\frac{1}{\kappa }+\frac{n_{I}+1}{2n_{I}}.\frac{1}{\delta }+\frac{1}{c_{h}+d_{inf}+\sigma \beta T_{0}} \end{array}$$ (4)

### Dose-response relationship in RVFV mosquito vectors

A systematic review of the literature was performed to study *F*(*V*), the relationship between a vertebrate host RVFV infectious titer and the associated probability to infect a mosquito upon its bite (Section S.2.1 in S1 Text). We limited our quantitative analysis to experiments performed with *Aedes* and *Culex* spp., with strain ZH501, on hamsters (Section S.2.1 in S1 Text). This corresponded to 185 data points from 9 papers.

To assess the impact of the diversity of protocols from which the data originated, we tested the effect of temperature, and number of days between mosquito feeding and dissection, in addition to dose (log_10_ infectious titer) on infection rates (presence of RVFV in the body of mosquitoes, legs excluded, Sections S.2.2, S.2.3 in S1 Text). For that we used a logistic function (Eq (S.5) in S1 Text), fitted with a binomial and a beta-binomial likelihood, the latter to account for overdispersal in the data (Section S.2.3 in S1 Text).

We used Akaike Information Criterion (AIC) to compare model fit of different functional forms (Section S.2.3 in S1 Text). Best fitting functions were then used to explore differences between and within genera (Table A and Fig G in S1 Text).

### Net infectiousness of RVFV livestock hosts

We define net infectiousness (NI) as the integral of an infectiousness curve over time (Eq (5))

$$\begin{array}{c} NI_{vect,host}=\int F_{vect}\left(V_{host}\left(t\right)\right)dt \end{array}$$ (5)

NI combines the dose-response relationship in vectors *F*_*vect*_(*V*) with infectious virus dynamics in hosts *V*_*host*_(*t*). As such, it must incorporate the uncertainty from both estimations. This was done by sampling 1000 parameter sets from *F*_*vect*_(*V*) and *V*_*host*_(*t*) respective fitting procedures. For lambs, a draw in a Bernoulli distribution first determined whether the viral load dynamics should be of a surviving or dying type. In the latter case, a time of death was sampled in a Weibull survival model fitted to death times present in our dataset, and determined the end of the viral load curve. Finally, a sensitivity analysis explored how the survival rate (probability of the Bernoulli sampling) in the lamb population impacts the average NI of lambs.

This quantity NI is proportional to the expected number of mosquitoes infected by a host over the entire course of its infection, assuming that biting occurs at a constant rate over this period. By extension, the NI ratio of two host categories is identical to the ratio of the expected number of mosquitoes infected by those two types of hosts, assuming bites to be equally distributed over both species. In the present study, NI was also vector-specific.

## Supporting information

S1 Text Supplemental details on results. **Fig A:** Likelihood profiles to estimate *T*_0_. **Fig B:** Trace plots of selected models. **Fig C:** Gelman diagnostic plots, per parameter, for selected models. **Fig D:** Joint posterior distributions of parameters per selected model. **Fig E:** Pairwise correlation between estimated parameters, for each group. **Fig F:** Distribution of temperature, days post-exposure, and infectious titers, in experimental data retrieved from the systematic review, for *Aedes* and *Culex* spp. vectors. **Fig G:** Species-specific dose-response curves. **Fig H:** Net infectiousness of an average lamb in relation with the expected survival rate in the population, for transmission to *Aedes* and *Culex* spp. vectors. **Table A:** Number of datapoints available per vector species, retrieved from the systematic review.
(PDF)

## References

[1] Taylor LH, Latham SM, woolhouse MEJ. Risk factors for human disease emergence. Phil Trans R Soc Lond B. 2001;356(1411):983–989. doi: 10.1098/rstb.2001.0888 11516376 PMC1088493

[2] Cleaveland S, Laurenson MK, Taylor LH. Diseases of humans and their domestic mammals: pathogen characteristics, host range and the risk of emergence. Phil Trans R Soc Lond B. 2001;356(1411):991–999. doi: 10.1098/rstb.2001.0889 11516377 PMC1088494

[3] Hollingsworth TD, Pulliam JRC, Funk S, Truscott JE, Isham V, Lloyd AL. Seven challenges for modelling indirect transmission: Vector-borne diseases, macroparasites and neglected tropical diseases. Epidemics. 2015;10:16–20. doi: 10.1016/j.epidem.2014.08.007 25843376 PMC4383804

[4] Buhnerkempe MG, Roberts MG, Dobson AP, Heesterbeek H, Hudson PJ, Lloyd-Smith JO. Eight challenges in modelling disease ecology in multi-host, multi-agent systems. Epidemics. 2015;10:26–30. doi: 10.1016/j.epidem.2014.10.001 25843378 PMC4437722

[5] Lloyd-Smith JO, Funk S, McLean AR, Riley S, Wood JLN. Nine challenges in modelling the emergence of novel pathogens. Epidemics. 2015;10:35–39. doi: 10.1016/j.epidem.2014.09.002 25843380 PMC4715032

[6] Webster JP, Borlase A, Rudge JW. Who acquires infection from whom and how? Disentangling multi-host and multi-mode transmission dynamics in the ‘elimination’ era. Phil Trans R Soc B. 2017;372(1719):20160091. doi: 10.1098/rstb.2016.0091 28289259 PMC5352818

[7] Roche B, Eric Benbow M, Merritt R, Kimbirauskas R, McIntosh M, Small PLC, et al. Identifying the Achilles heel of multi-host pathogens: the concept of keystone ‘host’ species illustrated by *Mycobacterium ulcerans* transmission. Environ Res Lett. 2013;8(4):045009. doi: 10.1088/1748-9326/8/4/045009 24554969 PMC3925833

[8] Vazquez-Prokopec GM, Perkins TA, Waller LA, Lloyd AL, Reiner RC, Scott TW, et al. Coupled Heterogeneities and Their Impact on Parasite Transmission and Control. Trends in Parasitology. 2016;32(5):356–367. doi: 10.1016/j.pt.2016.01.001 26850821 PMC4851872

[9] Fenton A, Streicker DG, Petchey OL, Pedersen AB. Are All Hosts Created Equal? Partitioning Host Species Contributions to Parasite Persistence in Multihost Communities. The American Naturalist. 2015;186(5):610–622. doi: 10.1086/683173 26655774 PMC6542667

[10] Martin LB, Addison B, Bean AGD, Buchanan KL, Crino OL, Eastwood JR, et al. Extreme Competence: Keystone Hosts of Infections. Trends in Ecology & Evolution. 2019;34(4):303–314. doi: 10.1016/j.tree.2018.12.009 30704782 PMC7114649

[11] Althouse BM, Hanley KA. The tortoise or the hare? Impacts of within-host dynamics on transmission success of arthropod-borne viruses. Philosophical Transactions of the Royal Society B: Biological Sciences. 2015;370(1675):20140299. doi: 10.1098/rstb.2014.0299 26150665 PMC4528497

[12] ten Bosch QA, Clapham HE, Lambrechts L, Duong V, Buchy P, Althouse BM, et al. Contributions from the silent majority dominate dengue virus transmission. PLOS Pathogens. 2018;14(5):e1006965. doi: 10.1371/journal.ppat.1006965 29723307 PMC5933708

[13] Kain MP, Bolker BM. Predicting West Nile virus transmission in North American bird communities using phylogenetic mixed effects models and eBird citizen science data. Parasites Vectors. 2019;12(1):395. doi: 10.1186/s13071-019-3656-8 31395085 PMC6686473

[14] Clapham HE, Tricou V, Van Vinh Chau N, Simmons CP, Ferguson NM. Within-host viral dynamics of dengue serotype 1 infection. Journal of The Royal Society Interface. 2014;11(96):20140094. doi: 10.1098/rsif.2014.0094 24829280 PMC4032531

[15] Ben-Shachar R, Koelle K. Minimal within-host dengue models highlight the specific roles of the immune response in primary and secondary dengue infections. Journal of The Royal Society Interface. 2015;12(103):20140886. doi: 10.1098/rsif.2014.0886 25519990 PMC4305404

[16] Ben-Shachar R, Schmidler S, Koelle K. Drivers of Inter-individual Variation in Dengue Viral Load Dynamics. PLOS Computational Biology. 2016;12(11):e1005194. doi: 10.1371/journal.pcbi.1005194 27855153 PMC5113863

[17] Clapham HE, Quyen TH, Kien DTH, Dorigatti I, Simmons CP, Ferguson NM. Modelling Virus and Antibody Dynamics during Dengue Virus Infection Suggests a Role for Antibody in Virus Clearance. PLOS Computational Biology. 2016;12(5):e1004951. doi: 10.1371/journal.pcbi.1004951 27213681 PMC4877086

[18] Koelle K, Farrell AP, Brooke CB, Ke R. Within-host infectious disease models accommodating cellular coinfection, with an application to influenza†. Virus Evolution. 2019;5(2). doi: 10.1093/ve/vez018 31304043 PMC6613536

[19] Schulze-Horsel J, Schulze M, Agalaridis G, Genzel Y, Reichl U. Infection dynamics and virus-induced apoptosis in cell culture-based influenza vaccine production—Flow cytometry and mathematical modeling. Vaccine. 2009;27(20):2712–2722. doi: 10.1016/j.vaccine.2009.02.027 19428884

[20] Pinilla LT, Holder BP, Abed Y, Boivin G, Beauchemin CAA. The H275Y Neuraminidase Mutation of the Pandemic A/H1N1 Influenza Virus Lengthens the Eclipse Phase and Reduces Viral Output of Infected Cells, Potentially Compromising Fitness in Ferrets. Journal of Virology. 2012;86(19):10651–10660. doi: 10.1128/JVI.07244-11 22837199 PMC3457267

[21] Petrie SM, Guarnaccia T, Laurie KL, Hurt AC, McVernon J, McCaw JM. Reducing Uncertainty in Within-Host Parameter Estimates of Influenza Infection by Measuring Both Infectious and Total Viral Load. PLoS ONE. 2013;8(5):e64098. doi: 10.1371/journal.pone.0064098 23691157 PMC3655064

[22] Petrie SM, Butler J, Barr IG, McVernon J, Hurt AC, McCaw JM. Quantifying relative within-host replication fitness in influenza virus competition experiments. Journal of Theoretical Biology. 2015;382:259–271. doi: 10.1016/j.jtbi.2015.07.003 26188087

[23] Simon PF, de La Vega MA, Paradis E, Mendoza E, Coombs KM, Kobasa D, et al. Avian influenza viruses that cause highly virulent infections in humans exhibit distinct replicative properties in contrast to human H1N1 viruses. Scientific Reports. 2016;6(1). doi: 10.1038/srep24154 27080193 PMC4832183

[24] Yan AWC, Zhou J, Beauchemin CAA, Russell CA, Barclay WS, Riley S. Quantifying mechanistic traits of influenza viral dynamics using in vitro data. Epidemics. 2020;33:100406. doi: 10.1016/j.epidem.2020.100406 33096342

[25] Daubney R, Hudson JR, Garnham PC. Enzootic hepatitis or rift valley fever. An undescribed virus disease of sheep cattle and man from east africa. The Journal of Pathology and Bacteriology. 1931;34(4):545–579. doi: 10.1002/path.1700340418

[26] Nanyingi MO, Munyua P, Kiama SG, Muchemi GM, Thumbi SM, Bitek AO, et al. A systematic review of Rift Valley Fever epidemiology 1931–2014. Infection Ecology & Epidemiology. 2015;5(1):28024. doi: 10.3402/iee.v5.28024 26234531 PMC4522434

[27] Al-Afaleq AI, Hussein MF. The Status of Rift Valley Fever in Animals in Saudi Arabia: A Mini Review. Vector-Borne and Zoonotic Diseases. 2011;11(12):1513–1520. doi: 10.1089/vbz.2010.0245 21923257

[28] El Mamy ABO, Baba MO, Barry Y, Isselmou K, Dia ML, Hampate B, et al. Unexpected Rift Valley Fever Outbreak, Northern Mauritania. Emerging Infectious Diseases. 2011;17(10):1894–1896. doi: 10.3201/eid1710.110397 22000364 PMC3310676

[29] LaBeaud AD, Kazura JW, King CH. Advances in Rift Valley fever research: insights for disease prevention. Current Opinion in Infectious Diseases. 2010;23(5):403–408. doi: 10.1097/QCO.0b013e32833c3da6 20613512 PMC3126654

[30] Linthicum KJ, Britch SC, Anyamba A. Rift Valley Fever: An Emerging Mosquito-Borne Disease. Annual Review of Entomology. 2016;61(1):395–415. doi: 10.1146/annurev-ento-010715-023819 26982443

[31] Bird BH, Ksiazek TG, Nichol ST, MacLachlan NJ. Rift Valley fever virus. 2009;234(7):11. 10.2460/javma.234.7.883 19335238

[32] Clark MHA, Warimwe GM, Di Nardo A, Lyons NA, Gubbins S. Systematic literature review of Rift Valley fever virus seroprevalence in livestock, wildlife and humans in Africa from 1968 to 2016. PLOS Neglected Tropical Diseases. 2018;12(7):e0006627. doi: 10.1371/journal.pntd.0006627 30036382 PMC6072204

[33] Bron GM, Strimbu K, Cecilia H, Lerch A, Moore S, Tran Q, et al. Over 100 years of Rift Valley Fever: a patchwork of data on pathogen spread and spillover. Pathogens. 2021;10(708). doi: 10.3390/pathogens10060708 34198898 PMC8227530

[34] Wichgers Schreur PJ, Oreshkova N, van Keulen L, Kant J, van de Water S, Soós P, et al. Safety and efficacy of four-segmented Rift Valley fever virus in young sheep, goats and cattle. npj Vaccines. 2020;5(1). 10.1038/s41541-020-00212-4 PMC7382487 32728479

[35] Quigley JD, Drewry JJ, Martin KR. Estimation of Plasma Volume in Holstein and Jersey Calves. Journal of Dairy Science. 1998;81(5):1308–1312. doi: 10.3168/jds.S0022-0302(98)75693-0 9621233

[36] Courtice FC. The blood volume of normal animals. The Journal of Physiology. 1943;102(3):290–305. doi: 10.1113/jphysiol.1943.sp004035 16991609 PMC1393413

[37] Coghlan JP, Fan JS, Scoggins BA, Shulkes AA. Measurement of extracellular fluid volume and blood volume in sheep. Aust J Biol Sci. 1977;30(1-2):71–84. doi: 10.1071/BI9770071 901309

[38] Krylova O, Earn DJD. Effects of the infectious period distribution on predicted transitions in childhood disease dynamics. Journal of The Royal Society Interface. 2013;10(84):20130098. doi: 10.1098/rsif.2013.0098 23676892 PMC3673147

[39] Lloyd AL. Realistic Distributions of Infectious Periods in Epidemic Models: Changing Patterns of Persistence and Dynamics. Theoretical Population Biology. 2001;60(1):59–71. doi: 10.1006/tpbi.2001.1525 11589638

[40] Canini L, Woolhouse MEJ, Maines TR, Carrat F. Heterogeneous shedding of influenza by human subjects and its implications for epidemiology and control. Scientific Reports. 2016;6(1). doi: 10.1038/srep38749 27966651 PMC5155248

[41] Smith AM, Adler FR, Perelson AS. An accurate two-phase approximate solution to an acute viral infection model. Journal of Mathematical Biology. 2010;60(5):711–726. doi: 10.1007/s00285-009-0281-8 19633852 PMC2841722

[42] Kruschke J. Doing Bayesian Data Analysis: A tutorial with R and Bugs. In: Doing Bayesian Data Analysis. Elsevier; 2015. p. i–ii. Available from: https://linkinghub.elsevier.com/retrieve/pii/B9780124058880099992.

[43] Ferguson NM, Hue Kien DT, Clapham H, Aguas R, Trung VT, Bich Chau TN, et al. Modeling the impact on virus transmission of *Wolbachia* -mediated blocking of dengue virus infection of *Aedes aegypti*. Sci Transl Med. 2015;7(279):279ra37–279ra37. doi: 10.1126/scitranslmed.3010370 25787763 PMC4390297

[44] Iwami S, Holder BP, Beauchemin CA, Morita S, Tada T, Sato K, et al. Quantification system for the viral dynamics of a highly pathogenic simian/human immunodeficiency virus based on an in vitro experiment and a mathematical model. 2012; p. 12. 10.1186/1742-4690-9-18 PMC3305505 22364292

[45] Vloet RPM, Vogels CBF, Koenraadt CJM, Pijlman GP, Eiden M, Gonzales JL, et al. Transmission of Rift Valley fever virus from European-breed lambs to Culex pipiens mosquitoes. PLOS Neglected Tropical Diseases. 2017;11(12):e0006145. doi: 10.1371/journal.pntd.0006145 29281642 PMC5760105

[46] Fontaine A, Lequime S, Moltini-Conclois I, Jiolle D, Leparc-Goffart I, Reiner RC, et al. Epidemiological significance of dengue virus genetic variation in mosquito infection dynamics. PLOS Pathogens. 2018;14(7):e1007187. doi: 10.1371/journal.ppat.1007187 30005085 PMC6059494

[47] Wichgers Schreur PJ, Vloet RPM, Kant J, van Keulen L, Gonzales JL, Visser TM, et al. Reproducing the Rift Valley fever virus mosquito-lamb-mosquito transmission cycle. Sci Rep. 2021;11(1):1477. doi: 10.1038/s41598-020-79267-1 33446733 PMC7809480

[48] Kroeker AL, Babiuk S, Pickering BS, Richt JA, Wilson WC. Livestock Challenge Models of Rift Valley Fever for Agricultural Vaccine Testing. Front Vet Sci. 2020;7:238. doi: 10.3389/fvets.2020.00238 32528981 PMC7266933

[49] Tuncer N, Gulbudak H, Cannataro VL, Martcheva M. Structural and Practical Identifiability Issues of Immuno-Epidemiological Vector–Host Models with Application to Rift Valley Fever. Bulletin of Mathematical Biology. 2016;78(9):1796–1827. doi: 10.1007/s11538-016-0200-2 27651156

[50] Tesla B, Demakovsky LR, Packiam HS, Mordecai EA. Estimating the effects of variation in viremia on mosquito susceptibility, infectiousness, and R0 of Zika in Aedes aegypti. PLOS Neglected Tropical Diseases. 2018;12(8):19. doi: 10.1371/journal.pntd.0006733 30133450 PMC6122838

[51] Elliott R, Weber F. Bunyaviruses and the Type I Interferon System. Viruses. 2009;1(3):1003–1021. doi: 10.3390/v1031003 21994579 PMC3185543

[52] Mapder T, Clifford S, Aaskov J, Burrage K. A population of bang-bang switches of defective interfering particles makes within-host dynamics of dengue virus controllable. PLOS Computational Biology. 2019;15(11):e1006668. doi: 10.1371/journal.pcbi.1006668 31710599 PMC6872170

[53] Jacobs NT, Onuoha NO, Antia A, Steel J, Antia R, Lowen AC. Incomplete influenza A virus genomes occur frequently but are readily complemented during localized viral spread. Nature Communications. 2019;10(1). doi: 10.1038/s41467-019-11428-x 31387995 PMC6684657

[54] Bermúdez-Méndez E, Katrukha EA, Spruit CM, Kortekaas J, Wichgers Schreur PJ. Visualizing the ribonucleoprotein content of single bunyavirus virions reveals more efficient genome packaging in the arthropod host. Commun Biol. 2021;4(1):345. doi: 10.1038/s42003-021-01821-y 33753850 PMC7985392

[55] Conti B, Tabarean I, Andrei C, Bartfai T. Cytokines and fever. Frontiers in Bioscience. 2004;9:1433–1449. doi: 10.2741/1341 14977558

[56] Wichgers Schreur PJ, van Keulen L, Kant J, Oreshkova N, Moormann RJM, Kortekaas J. Co-housing of Rift Valley Fever Virus Infected Lambs with Immunocompetent or Immunosuppressed Lambs Does Not Result in Virus Transmission. Front Microbiol. 2016;7. doi: 10.3389/fmicb.2016.00287 27014211 PMC4779905

[57] Lequime S, Dehecq JS, Matheus S, de Laval F, Almeras L, Briolant S, et al. Modeling intra-mosquito dynamics of Zika virus and its dose-dependence confirms the low epidemic potential of Aedes albopictus. PLOS Pathogens. 2020;16(12):e1009068. doi: 10.1371/journal.ppat.1009068 33382858 PMC7774846

[58] Weaver SC, Forrester NL, Liu J, Vasilakis N. Population bottlenecks and founder effects: implications for mosquito-borne arboviral emergence. Nat Rev Microbiol. 2021;19(3):184–195. doi: 10.1038/s41579-020-00482-8 33432235 PMC7798019

[59] Bustamante DM, Lord CC. Sources of Error in the Estimation of Mosquito Infection Rates Used to Assess Risk of Arbovirus Transmission. American Journal of Tropical Medicine and Hygiene. 2010;82(6):1172–1184. doi: 10.4269/ajtmh.2010.09-0323 20519620 PMC2877431

[60] Golnar AJ, Turell MJ, LaBeaud AD, Kading RC, Hamer GL. Predicting the Mosquito Species and Vertebrate Species Involved in the Theoretical Transmission of Rift Valley Fever Virus in the United States. PLoS Neglected Tropical Diseases. 2014;8(9):e3163. doi: 10.1371/journal.pntd.0003163 25211133 PMC4161329

[61] Turell MJ, Dohm DJ, Mores CN, Terracina L, Wallette DL, Hribar LJ, et al. Potential for North American Mosquitoes to Transmit Rift Valley Fever Virus ^1^. Journal of the American Mosquito Control Association. 2008;24(4):502–507. doi: 10.2987/08-5791.1 19181056

[62] Turell MJ, Wilson WC, Bennett KE. Potential for North American Mosquitoes (Diptera: Culicidae) to Transmit Rift Valley Fever Virus. J Med Entomol. 2010;47(5):884–889. doi: 10.1093/jmedent/47.5.884 20939385

[63] Turell MJ, Britch SC, Aldridge RL, Kline DL, Boohene C, Linthicum KJ. Potential for Mosquitoes (Diptera: Culicidae) From Florida to Transmit Rift Valley Fever Virus. J Med Entomol. 2013;50(5):1111–1117. doi: 10.1093/jme/tjv093 24180117

[64] Turell MJ, Cohnstaedt LW, Wilson WC. Effect of Environmental Temperature on the Ability of *Culex tarsalis* and *Aedes taeniorhynchus* (Diptera: Culicidae) to Transmit Rift Valley Fever Virus. Vector-Borne and Zoonotic Diseases. 2020;20(6):454–460. doi: 10.1089/vbz.2019.2554 32017863

[65] Gog JR, Pellis L, Wood JLN, McLean AR, Arinaminpathy N, Lloyd-Smith JO. Seven challenges in modeling pathogen dynamics within-host and across scales. Epidemics. 2015;10:45–48. doi: 10.1016/j.epidem.2014.09.009 25843382

[66] Beauchemin CA, Handel A. A review of mathematical models of influenza A infections within a host or cell culture: lessons learned and challenges ahead. BMC Public Health. 2011;11(Suppl 1):S7. doi: 10.1186/1471-2458-11-S1-S7 21356136 PMC3317582

[67] Roberts GO, Gelman A, Gilks WR. Weak convergence and optimal scaling of random walk Metropolis algorithms. The Annals of Applied Probability. 1997;7(1):110–120.

[68] Miao H, Xia X, Perelson AS, Wu H. On Identifiability of Nonlinear ODE Models and Applications in Viral Dynamics. SIAM Review. 2011;53(1):3–39. doi: 10.1137/090757009 21785515 PMC3140286

[69] Beauchemin CAA, McSharry JJ, Drusano GL, Nguyen JT, Went GT, Ribeiro RM, et al. Modeling amantadine treatment of influenza A virus in vitro. Journal of Theoretical Biology. 2008;254(2):439–451. doi: 10.1016/j.jtbi.2008.05.031 18653201 PMC2663526

[70] Wallinga J, Lipsitch M. How generation intervals shape the relationship between growth rates and reproductive numbers. Proc R Soc B. 2007;274(1609):599–604. doi: 10.1098/rspb.2006.3754 17476782 PMC1766383

[71] Svensson A. A note on generation times in epidemic models. Mathematical Biosciences. 2007;208(1):300–311. doi: 10.1016/j.mbs.2006.10.010 17174352

