# Supplementary material for: Heterogeneity of Rift Valley fever virus transmission potential across livestock hosts, quantified through a model-based analysis of host viral load and vector infection
Source: PLoS Comput Biol. 2022 Jul 22;18(7):e1010314. doi: 10.1371/journal.pcbi.1010314 (PMC9348665; doi:10.1371/journal.pcbi.1010314)
Supplement: Attachment — Submitted filename: PCOMPBIOL-D-21-02043_response_letter.odt [file pcbi.1010314.s002.odt]

## PCOMPBIOL-D-21-02043

Heterogeneity of Rift Valley fever virus transmission potential across livestock hosts, quantified through a model-based analysis of host viral load and vector infection

First of all, we would like to thank the two referees for their helpful comments on our manuscript. We have addressed them in the new version of the manuscript. Please find thereafter in blue our response to these comments, also indicating where the manuscript has been modified (line numbers in the files with track changes).
We point out that 3 new papers were added to the reference list:

Conti, B., I. Tabarean, C. Andrei, and T. Bartfai (2004). “Cytokines and fever”. In: Frontiers in Bioscience 9, pp. 1433–1449.

Wichgers Schreur, P. J., L. van Keulen, J. Kant, N. Oreshkova, R. J. M. Moormann, and J. Kortekaas (2016). “Co-housing of Rift Valley Fever Virus Infected Lambs with Immunocompetent or Immunosuppressed Lambs Does Not Result in Virus Transmission”. en. In: Front. Microbiol. 7. doi: 10.3389/fmicb.2016.00287.

Kroeker, A. L., S. Babiuk, B. S. Pickering, J. A. Richt, and W. C. Wilson (2020). “Livestock Challenge Models of Rift Valley Fever for Agricultural Vaccine Testing”. en. In: Front. Vet. Sci. 7, p. 238. doi: 10.3389/fvets.2020.00238.

the authors mention that the log-likelihood was "similar to Clapham et al (2014)" but don't seem to specify it explicitly in the main text or SI. Please give the likelihood in the methods section.
It now appears as Equation 2 in main text, with detailed explanations l.297-306.
“In our composite log-likelihood f (Eq. 2), we assumed log10 viremia measurements had normally distributed errors. Below, φ and Φ are respectively the probability and cumulative density functions of the normal distribution, and ε2 is taken to be 1 (Clapham et al. 2014). D is the measure of either viral RNA or infectious viruses (subscript i), and x the associated model prediction (either Vtot or Vinf). Measures below the limit of detection (LOD) are considered to be at or below LOD (Clapham et al. 2014). The final log-likelihood of a given model parametrization is the sum across types of measures i, timesteps j, and individuals k of the group under consideration.” 

Reviewer #1: The authors have created a nice within-host model that allows them to evaluate variability between hosts in infectious period, and likelihood of infecting a mosquito. My concerns primarily have to do with better documenting the biological methods.

R1.0 It appears that the data driving the estimates used in this model were obtained from a study of vaccinations. The authors cite the article (Safety and efficacy of four segmented RVFV in young sheep, goats, cattle) for all of their biological methods. I would suggest a brief description in these methods as well - so that a reader at least understands the study design (like fiigure 4 in the citation) and how TCID/viral burden (methods sections in the citation were titled RTqPCR and viral isolation) was calculated since those are the parameters that are important in their model.
Although the data used in this study are obtained from studies in which a candidate vaccine was tested, we here only used the data of the positive control groups, which were mock vaccinated. We acknowledge that a bit more detail about these groups would be favourable and added extra details to section 4.1. Furthermore we added more details about the methods of the RT-qPCR and TCID50 measurements. Note that sections related to data in results (2.2) and material and methods (4.1) were rearranged as advised in comment R.2.1. 

l.249-250 “Plasma was sampled daily for 10 days in goats, daily for 9 days then every two days until day 14 in calves and lambs.”
 
l.257-268 “Viral RNA was isolated with the NucliSENS easyMAG system according the manufacturer’s instructions (bioMerieux, France) from either 0.5 ml of plasma or 0.5 ml of 10% organ suspension. Briefly, 5 µl RNA was used in a RVFV RT-qPCR using the LightCycler one-tube RNA Amplification Kit HybProbe (Roche, Almere, The Netherlands) in combination with a LightCycler 480 real-time PCR system (Roche) and the RVS forward primers (AAAGGAACAATGGACTCTGGTCA), the RVAs (CACTTCTTACTACCATGTCCTCCAAT) reverse primer and a FAM-labelled probe RVP (AAAGCTTTGATATCTCTCAGTGCCCCAA)25. Virus isolation was performed on RT-qPCR positive samples with a threshold above 105 RNA copies/ml as this has been previously shown to be a cut-off point below which no live virus can be isolated. For the virus isolations, plasma or 10% organ suspensions were used. Briefly, BHK-21 cells were seeded at a density of 20,000 cells/well in 96-well plates. Serial dilutions of samples were incubated with the cells for 1.5 h before medium replacement. Cytopathic effect was evaluated after 5–7 days post infection and tissue culture infective dose 50 (TCID50) was calculated using the Spearman–Kärber algorithm.”

R1.1 As a note - the citations - Wichgers 2020a and 2020b appear to be the same citation. Was this accidental? Was one supposed to be a different citation?
This was indeed a mistake. We corrected it (one unique paper referenced l.76, 248, 254).

R1.2 Additionally the authors rightly mention in their discussion that taking into account host immunity would be very important in future iterations. I understand the authors may not want to alter their model for this publication but they do have two very easy to use parameters in the study design if i read the citation correctly - neutralizing antibody titers and body temperature. It would be interesting to specifically mention how they might incorporate those in the discussion.
Regarding neutralizing antibodies, these were only measured at day 7 post-infection, when infectious viruses are no longer detectable and viral RNA has already dropped significantly. This type of data was therefore of limited utility for the present model, as innate immune response is primarily involved in controlling viremia for primary infections.
As with temperature, it could indeed be considered as a proxy of the cytokine storm, and we did think of incorporating it as input data, to drive the variation of some model parameters over time. However, which parameter(s) should have been impacted, and with what kind of functional form, is a different research question than the one adressed in our paper and should be studied separately. We added in the discussion, l.203-207 “Alternatively, routinely collected data such as body temperature could constitute an interesting lead to explore time-varying parameters, as a proxy of the immune response. Dedicated modeling would first be needed to determine i) the form of the relationship between temperature and the immune response, most likely with cytokines (Conti et al. 2004, Wichgers Schreur et al. 2016), and ii) which model parameters should be impacted by such an immune response.”

R1.3 And lastly it appears the work was done on 1 breed of goat, 1 of cattle and 1 of sheep. And each of those species was of approximately different age. Do the authors think that other breeds of the same species would have any alterations? They should at least mention that they can't be sure that the estimates for sheep would be relevant to all sheep breeds, goat breeds, cattle breeds. Some breeds are bred to be disease resistant and so might have altered estimates. Additionally the authors should mention that the animals were of different ages and clarify whether this is important in their conclusions at all?
Regarding animal breed, we precised our previous statement l.175-179 “The breeds infected in our dataset, which are dominant breeds from Europe, make our estimates directly relevant for scenarios of RVFV emergence in this continent. A comparison with African breeds is required to know if the relative differences in infectiousness are maintained.” 

Regarding animal’s age, sheep and goats needed not to rely on milk from the mothers anymore. 
For calves (because they can drink artificial milk) we could use younger animals, which are smaller and also easier to handle. Lambs were below 3 month old, and it has been highlighted that the drop in severity happens in 4-6 month old sheep (Kroeker et al. 2020 doi: 10.3389/fvets.2020.00, Table 2). Still, high viremia can be seen in older individuals too (Kroeker et al. 2020, Table 2). Overall, all ruminants below 3 month old are highly susceptible to RVF (Kroeker et al. 2020). We precised our previous statement l.184-186 “Quantifying more precisely the effect of aging on animals’ viral dynamics and pathogenesis is needed to complete our results (Kroeker et al. 2020).”

Reviewer #2:
The study investigates the within-host infection dynamics and their epidemiological consequences of Rift Valley fever virus with an objective to understand the relative importance of livestock species in disease transmission. By fitting a mathematical model of within-host infection to experimental data from relevant livestock species, the authors compare the basic reproductive number and generation time between them. The authors then carried out a literature review to parameterise the relationship between infection load and per capita transmissibility. I find the study to be comprehensive and methodologically sound. The manuscript is concise and well written. Below I list my minor comments.

R2.0 Given the audience of PLOS Comp Biol, I believe that the abstract should start off a little more general (i.e., the first sentence should introduce the concept, not the studied virus). I think the authors already do a great job at this in their Introduction.
We added the following sentences at the beginning of the abstract: “Quantifying the variation of pathogens’ life history traits in multiple host systems is crucial to understand their transmission dynamics. This is particularly important for arthropod-borne viruses (arboviruses), which are prone to infecting several species of vertebrate hosts. Here, we focus on how host-pathogen interactions determine the ability of host species to transmit a virus to susceptible vectors upon a potentially infectious contact.”

R2.1 I found the structure of the paper a little strange, with a subsection called “2.2. Data” appearing under the Results section. I recommend merging “2.2. Data” with “4.1. Data”.
We think both sections serve their own purpose. A small descriptives of the data is needed before the modeling results to understand the overall dynamics measured. The part in the methods section needs to include the relevant aspects of the experimental design so that readers do not necessarily need to read the associated publication in full, and was modified following comment R.1.0. We made the following changes, making sure results and methods do not overlap, which was confusing before.
in “2.1 Overall approach” l.74-76
We fitted this model to time-series of viral RNA (RT-qPCR) and infectious virus (TCID50), measured daily in calves (n=8), lambs (n=16), and young goats (n=8) intravenously inoculated with a virulent RVFV strain (Wichgers Schreur et al., 2020, Section 4.1).

in Results – section now called “2.2 Data description” l.86-91
All animals became viremic. In total, 10/16 lambs succumbed to the infection or were euthanized, 3 to 7 days after RVFV inoculation, while others survived until the end of the experiment (2 weeks). All calves and young goats survived until the end of the experiment. Animals reached their maximum RNA levels (average 8.79 log10/ml, standard deviation 0.81 log10/ml) and infectious titers (average 5.16 log10/ml, standard deviation 1.16 log10/ml) on day 2 or 3 post infection.

in M&M – section now called “4.1 Experimental design” l.247-268
Data on viral RNA and infectious virus were obtained from a published study on a candidate RVFV vaccine (Wichgers Schreur et al., 2020). Mock vaccinated animals (8 lambs, 8 calves, 8 young goats) were inoculated intravenously with 5 log10 TCID50 of strain rRVFV 35/74. Plasma was sampled daily for 10 days in goats, daily for 9 days then every two days until day 14 in calves and lambs. Animals’ age was 2-3 weeks for calves, 8-10 weeks for lambs and goats. The average body weight of animals, used further to calibrate the inoculum per ml of plasma, was 45 kg for lambs, 30 kg for goats, and 80 kg for calves. Animals were purchased from conventional Dutch farms, and the breed was Texel cross for sheep, Saanen for goats, and Holstein-Friesian for cattle (Wichgers Schreur et al., 2020b). An additional dataset obtained from 8 lambs, following the same protocol, was added. 

Viral RNA was isolated with the NucliSENS easyMAG system according the manufacturer’s instructions (bioMerieux, France) from either 0.5 ml of plasma or 0.5 ml of 10% organ suspension. Briefly, 5 µl RNA was used in a RVFV RT-qPCR using the LightCycler one-tube RNA Amplification Kit HybProbe (Roche, Almere, The Netherlands) in combination with a LightCycler 480 real-time PCR system (Roche) and the RVS forward primers (AAAGGAACAATGGACTCTGGTCA), the RVAs (CACTTCTTACTACCATGTCCTCCAAT) reverse primer and a FAM-labelled probe RVP (AAAGCTTTGATATCTCTCAGTGCCCCAA)25. Virus isolation was performed on RT-qPCR positive samples with a threshold above 105 RNA copies/ml as this has been previously shown to be a cut-off point below which no live virus can be isolated. For the virus isolations, plasma or 10% organ suspensions were used. Briefly, BHK-21 cells were seeded at a density of 20,000 cells/well in 96-well plates. Serial dilutions of samples were incubated with the cells for 1.5 h before medium replacement. Cytopathic effect was evaluated after 5–7 days post infection and tissue culture infective dose 50 (TCID50) was calculated using the Spearman–Kärber algorithm.

R2.2 Figure 2. The error bands capture the uncertainty from posterior distributions, but ignore the process of sampling. I recommend instead plotting predictive intervals that reflect both the posterior distributions and sampling errors of the data generating process (which is stated to be normally distributed in the Methods).
We modified Figure 2 and its legend accordingly. The median fit is now the median of the 1000 trajectories computed from posterior draws, and we show two envelopes : one for the uncertainty of the parameter estimation process, corresponding to the [2.5;97.5]% quantiles of the posterior trajectories, and one for the uncertainty of the sampling process, which we compute by adding +-1.96 around to the median fit ([quantiles 2.5;97.5]% of a normal distribution with standard deviation of 1). All in log10 scale. This is asymptotically equivalent to adding a random noise sampled in this normal distribution to every datapoints of the posterior trajectories and selecting 95% of the results. This is now precised in Supplementary Information S.1.1.

R2.3 On page 5, the authors state that “highest density intervals (HDIs) for this parameter are wide (Table 1), but the posterior distributions remain informative (Figure S.4).” But it’s not clear what is meant by “the posterior distributions remain informative”. It doesn’t appear to be a claim about the relative information between the prior and observations as I could not find an assessment for example of posterior contraction in the paper. (sensu https://betanalpha.github.io/assets/case_studies/principled_bayesian_workflow.html).
Although we did not quantify posterior contraction, the shape of the posterior (subplots “xi” of Figure S.4) is sufficient in our opinion to state that we gained knowledge compared to flat uniform distributions used as priors. To better visualize this, we have now set the x-axis limits in this figure to the width of the prior distributions. However, not knowing the true value of the parameter, we cannot know for sure if we are facing a case of overfitting, as shown in visual examples of section 1.3.2 of the link you provided. We clarified what we meant, l.108 “the posterior distributions remain informative, as knowledge was gained compared to uniform prior distributions”

R2.4 As within-host models are typically over-parameterised, the reader may benefit from understanding how the within-host parameters related and how much data can inform them. Pairwise plots of posterior draws are typically shown for visualising parameter collinearity.
We added pairwise correlations in Figure S.5. This figure is now referenced l.322.

R2.5 Figure 4. Posterior draws are falsely labelled as predictive intervals. I recommend calculating the quantiles. Please refer to my comment above in relation to Figure 2.
We changed Figure 4 and the wording accordingly. Indeed we did not have posteriors as we were not using a bayesian approach for this fitting, therefore no prediction interval either. Confidence intervals are now shown, resulting from the variability in 1000 replicate trajectories. This is stated in the figure legend. We also clarified the numerical example given l.136-138 : “At 5 log10 TCID50/ml for instance, which most animals could reach or exceed, there is 25% [17 ; 37] probability to infect an Aedes spp. vector and 11% [7 ; 18] probability to infect a Culex spp. Vector (point estimate and 95% confidence interval)”. We also added in Supplementary Information S.2.3 “To quantify the uncertainty around fitted curves, we sampled 1000 parameter sets using multivariate normal sampling with the covariance matrix of the fitting procedure. The resulting 1000 trajectories were used to extract 95% confidence intervals.”

R2.6 I found a mixed-use of British and American spelling throughout.
We did our best to stick to US english and therefore switched viraemic/viraemia into viremic/viremia and modelling into modeling throughout.


Lastly, we want to point out changes we’ve made independently of reviewers’ comments : 
The L state in the model diagram (Figure 1) was changed from “latently infected” to “latent state”, to clarify that it was meant as a modeling choice to introduce delay in virion production rather than a true latent infection of cells, which is not known for RVFV.
We have changed our wording in the way we present our results on dose-response relationships in Aedes and Culex, to clarify that this is not a general statement but an estimation based on currently available data in the literature, limited to some species of these genera.
